# Germinal and Somatic Trisomy 21 Mosaicism: How Common is it, What are the Implications for Individual Carriers and How Does it Come About?

**DOI:** 10.2174/138920210793176056

**Published:** 2010-09

**Authors:** Maj A. Hultén, Jon Jonasson, Ann Nordgren, Erik Iwarsson

**Affiliations:** 1 Warwick Medical School, University of Warwick, UK; 2 Department of Clinical and Experimental Medicine, Linköping University, Sweden; 3 Department of Molecular Medicine and Surgery, Karolinska Institutet, Sweden

**Keywords:** Trisomy 21, mosaicism, germ line, fetus, childhood leukemia, cancer, Alzheimer’s Disease.

## Abstract

It is well known that varying degrees of mosaicism for Trisomy 21, primarily a combination of normal and Trisomy 21 cells within individual tissues, may exist in the human population. This involves both Trisomy 21 mosaicism occurring in the germ line and Trisomy 21 mosaicism documented in different somatic tissues, or indeed a combination of both in the same subjects. Information on the incidence of Trisomy 21 mosaicism in different tissue samples from people with clinical features of Down syndrome as well as in the general population is, however, still limited. One of the main reasons for this lack of detailed knowledge is the technological problem of its identification, where in particular low grade/cryptic Trisomy 21 mosaicism, i.e. occurring in less than 3-5% of the respective tissues, can only be ascertained by fluorescence *in situ* hybridization (FISH) methods on large cell populations from the different tissue samples.

In this review we summarize current knowledge in this field with special reference to the question on the likely incidence of germinal and somatic Trisomy 21 mosaicism in the general population and its mechanisms of origin. We also highlight the reproductive and clinical implications of this type of aneuploidy mosaicism for individual carriers. We conclude that the risk of begetting a child with Trisomy 21 Down syndrome most likely is related to the incidence of Trisomy 21 cells in the germ line of any carrier parent. The clinical implications for individual carriers may likewise be dependent on the incidence of Trisomy 21 in the relevant somatic tissues. Remarkably, for example, there are indications that Trisomy 21 mosaicism will predispose carriers to conditions such as childhood leukemia and Alzheimer’s Disease but there is on the other hand a possibility that the risk of solid cancers may be substantially reduced.

## INTRODUCTION

Trisomy 21 (T21) associated with the clinical picture of Down syndrome (DS; OMIM90685) is the most common genetic cause of learning disability and congenital malformations in the general population. T21 is also a common cause of reproductive failure associated with miscarriage.

**Fig. (1):**
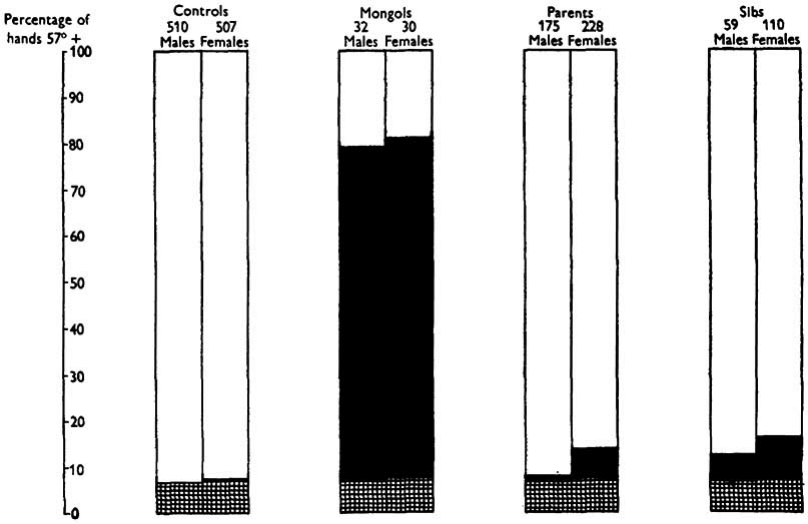
Diagram showing percentage incidence of the typical DS dermatoglyphics feature in different groups of subjects aged 15 years or over. The control population incidence is increased more than tenfold in DS cases, and is almost doubled in their mothers, brothers and sisters. The excess is shown by black rectangles. Reproduced from [8].

Many previous studies have highlighted the occurrence of T21 mosaicism in asymptomatic carriers as deduced primarily by investigation of *in vitro *cultured blood lymphocytes from parents and sibs of children with Down syndrome, implying both germinal and somatic T21 mosaicism [review in 1]. Remarkably, already before the discovery of T21 as the cause of DS *per se *in 1959, Penrose applied what we would now call a biomarker for identification of T21 mosaicism in parents and sibs of DS children, i.e. the typical dermatoglyphics that reflect embryological development (Fig. **1**) [2-8].

A number of different types of family studies indicate that a combination of germinal and somatic T21 mosaicism may be quite common in the general population. However, direct documentation of T21 in germinal cell populations *per se *has been rare by comparison. It would appear that the main reason for this lack of detailed information is the technological hurdles involved. One of the problems is that the only currently available approach for the detection of low grade/cryptic mosaicism (also termed micromosaicism) involves fluorescence *in situ *hybridization (FISH) applying at least two chromosome 21-specific probes together with a control probe [review in 9-19]. Furthermore, large-scale investigations on different germinal and somatic tissues present their own specific problems as regards access to and analysis of the relevant tissue samples.

We here summarize current knowledge in this field and highlight the reproductive and clinical implications of T21 mosaicism as well as its mechanisms of origin.

## RESULTS AND DISCUSSION

Very few direct investigations have so far been performed with a view to identify the incidence of T21 mosaicism in the germ line in relation to that in different somatic tissues. Whenever a larger number of cells have been studied in either situation the conclusion has been reached that T21 mosaicism is surprisingly common in the general population. Thus, it may in fact seem likely that T21 mosaicism is a biological feature shared in common between many, if not all people in the general population. Importantly, however, there is substantial variation in this character, both between individual subjects and between different tissues within individuals.

### Methodological Aspects

#### It is Only Fluorescence In Situ Hybridization (FISH) that Reveals Low Grade/Cryptic Mosaicism

Conventional cytogenetic technology, applied routinely in Genetics Service Laboratories for confirmation of the clinical diagnosis of Down syndrome provides only limited information with respect to T21 mosaicism [20]. In the majority of cases referred for this purpose only 10-15 cells in metaphase from *in vitro *cultured blood lymphocytes would be analyzed. In most cases having the typical DS phenotype only cells with the extra chromosome 21 would be seen, and it would then be concluded that this would be a case of so-called ‘complete’ T21. On the other hand, whenever one or two cells among the 10-15 would be found to have the normal chromosome constitution, the analysis would be extended to 50 cells or so. The generally accepted conclusion is that in the order of 1-5% of people clinically diagnosed as having DS are in fact ‘high grade’ T21 mosaics.

**Fig. (2):**
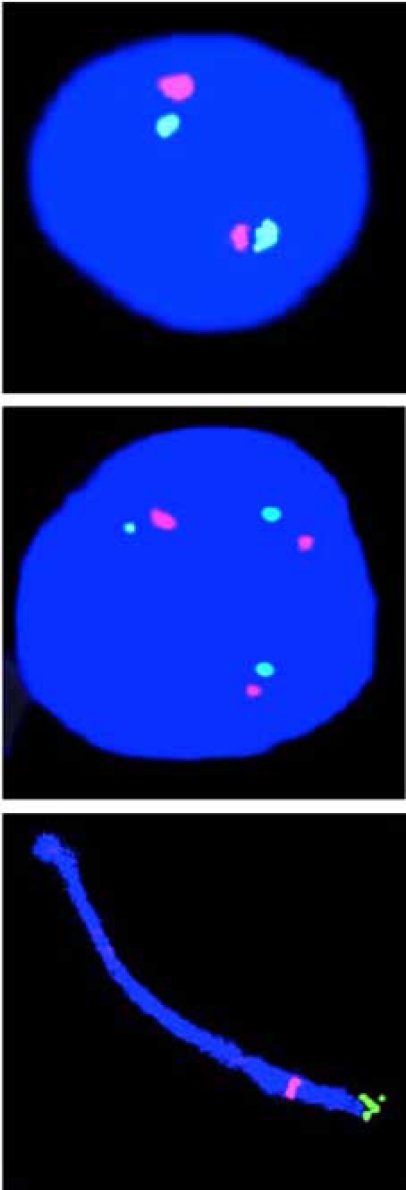
FISH images of fetal ovarian cell nuclei using two chromosome 21-specific probes located near the end of 21q (bottom), one normal disomy 21 nucleus (top) and one T21 cell nucleus (middle) illustrating female T21 germinal mosaicism. Reproduced from [24].

**Fig. (3):**
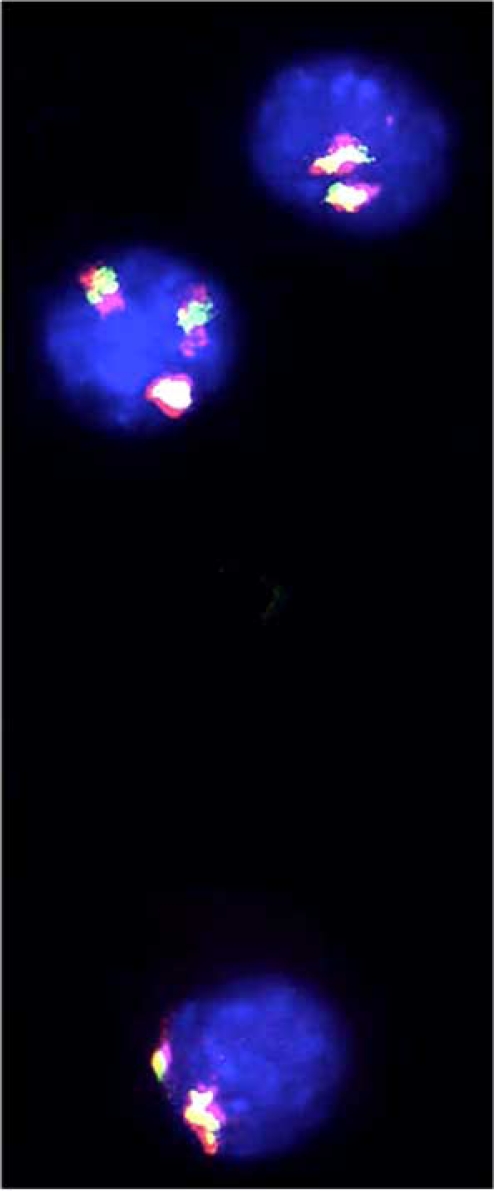
FISH images of two AD brain nuclei with disomy 21 (top and bottom) and a nucleus with T21 (middle) revealed by a chromosome 21-specific multicolor probe. Reproduced from [115].

Methodological limitations by way of the labor involved in the analysis of a large number of metaphases from PHA stimulated blood lymphocytes have hampered the identification of T21 micromosaics. The only technology that readily allows counts of chromosome copy number in large cell populations (in particular interphase nuclei) is FISH (Figs. **2,3**) first introduced into the Clinical Service in the 1990s [21, review in 9-19, 22, 23]. It is nevertheless important to recognize the limitations in accuracy of the FISH technology *per se*. The main complication in interpretation of chromosome copy number is the occurrence of false positive and false negative signals, unless at least two chromosome-specific probes (or so-called multicolor banding probes) are applied.

### Female Germinal Mosaicism

It comes as no surprise that few studies have addressed the question on the incidence of female germinal T21 mosaicism in the normal population, as this requires access to ovarian cells, which are not readily ascertained. Secondly, as already stressed, it is only FISH analysis of large populations of such cells that will inform on the occurrence of T21 micromosaicism. Three types of ovarian cell populations have been investigated in this respect, i.e. fetal cells obtained following termination of pregnancy for a non-medical/social reason, oocytes following *in vitro *fertilization and ovarian cells in ovarian biopsies from adult women [see Table 1 in 24].

#### Most Female Fetuses may be Low-Grade T21 Germinal Mosaics

As far as we are aware there is only one study documenting the incidence of T21 mosaicism in human fetal ovaries, using direct microscopy analysis recording the copy number of chromosome 21 by virtue of FISH analysis of individual cell nuclei [24]. Here FISH with two chromosome 21-specific probes was used to determine the copy number of chromosome 21 in ovarian cells from eight female fetuses at gestational age 14–22 weeks (Fig. **2**). All eight phenotypically normal female fetuses were found to be T21 mosaics, containing ovarian cells with an extra chromosome 21 (mean 0.54%, range 0.20-0.88%; SD 0.23).

#### Accumulation of T21 Oocytes During Development may Explain the Maternal T21 Age Effect

**Fig. (4):**
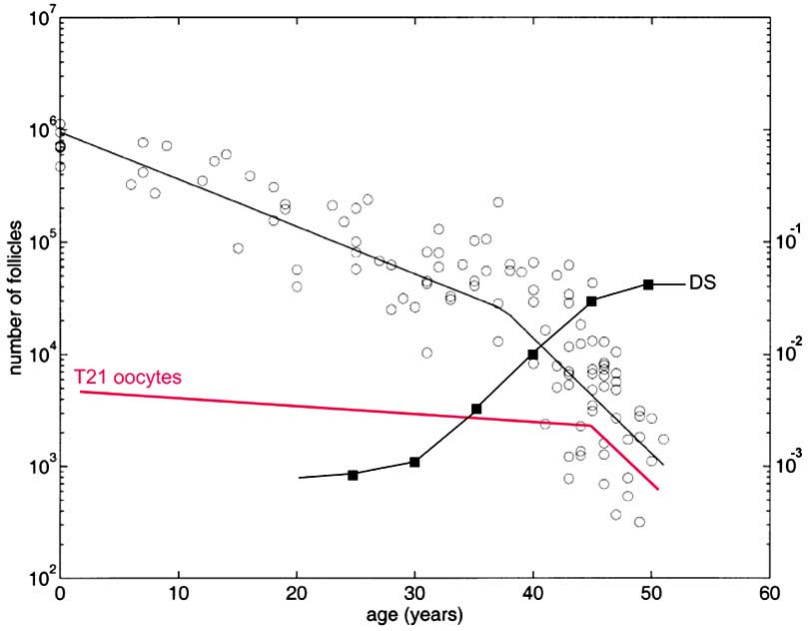
Increased proportion of T21 oocytes in the ageing ovary. The OMS hypothesis proposes that the T21 oocytes lag behind during development, resulting in higher proportions of the total oocyte pool over time. The figure illustrates the predicted number of T21 oocytes from birth until menopause (pink line) in comparison to the total (black circles) based on follicle counts (left hand Y axis) by Faddy [142]. The observed incidence (right hand Y axis) of T21 DS births (black squares) is represented by the data of Morris *et al.* [143]. The offset of the (pink) line showing the predicted number of T21 oocytes is based on the 0.54% mosaicism observed by Hultén *et al.* [24]. The slope is an approximation generating the expected DS birth rates with increasing maternal age. Note that the figure illustrates the principle of this hypothesis only and the lines drawn are based on rather uncertain estimates. Reproduced from [25].

Based on these observations, it is suggested that most normal female fetuses are T21 ovarian mosaics and the maternal age effect is caused by differential selection of these cells during fetal and postnatal development until ovulation (Fig. **4**) [24, 25]. Further studies are required to test this hypothesis by investigation of the relative frequency of T21 oocytes in fetal ovaries in relation to that in populations of oocytes at the Germinal Vesicle/Metaphase I stage, obtained from adult women of different biological age. It is further suggested that the exceptional occurrence of high-grade fetal germinal mosaicism may explain why young DS mothers have an increased risk in subsequent pregnancies [1, 26-29].

So far only a small number of studies have been performed on adult ovaries relevant to this question [Table 2 in 24]. The degree of T21 oocyte/ovarian mosaicism in these seven women, who all having had one ore more children with DS, varied substantially, i.e. between 5.71- and 94.00%. In this small sample, where oocytes/ovarian cells were analyzed directly by cytogenetic technology there was no correlation between the proportion of T21 oocytes/ovarian cells and number of previous children with DS (this number ranging from one to nine). A much larger number of cases of germinal (and somatic) T21 mosaicism has more recently been recorded, primarily because of reproductive history, i.e. one or more offspring with T21 [Table S1 in 1]. These data indicate that recurrence risk is related to maternal age, this risk being increased in younger mothers.

### Male Germinal T21 Mosaicism

The situation as regards male germinal T21 mosaicism is different and in a way more complex than that in females. Again, as far as we are aware there is only one study documenting the incidence of T21 mosaicism in human fetal testis, using direct microscopy analysis recording the copy number of chromosome 21 by virtue of FISH analysis of individual cell nuclei [30]. On the other hand, numerous studies have been performed documenting the incidence of disomy 21 in sperm, both from normally fertile males and males suffering from fertility problems [31-38].

#### Most Male Fetuses may Harbor Few if any T21 Cells in their Testes

FISH with two chromosome 21-specific probes has been used to determine the copy number of chromosome 21 in fetal testicular cell nuclei from four male fetuses, following termination of pregnancy for a non-medical/social reason at gestational age 14-19 weeks [30]. The cells studied were selected on the basis of their morphology alone, pending immunological specification of the relevant cell types. There was no indication of testicular T21 mosaicism in any of these four male fetuses, when analyzing at least 2000 cells per case (range 2038-3971, total 11,842). In a later extended study, two T21 cells in a total population of 20.000 fetal testicular cells were recorded, i.e. a frequency of 0.01% [Hultén *et al*., unpublished observations]. This result is highly statistically significant (p<0.001) in comparison to the average of 0.54% ovarian T21 mosaicism (range 0.20-0.88%) that was identified in eight female fetuses analyzing a total of 12,634 cells [24].

This observation suggests that there is a significant sex difference in degrees of fetal germinal T21 mosaicism. Thus, it would appear that most female fetuses are T21 ovarian mosaics, while in sharp contrast most male fetuses may be either very low grade T21 testicular mosaics or they may be non-mosaics. It is further proposed that this sex difference in germinal T21 mosaicism may explain the much less frequent paternal origin of T21 DS than maternal. The mechanisms underlying the DS cases, where the extra chromosome 21 does originate from the father (5-10%) remain unknown and further studies in this respect are required [30].

#### Most Men are Germinal chr21 Mosaics by way of Sperm Analysis

In contrast to the scarcity of studies investigating testicular T21 mosaicism, there are numerous investigations (to date totaling at least 34) recording the rate of disomy 21 in sperm from apparently normal controls. Results vary quite substantially in estimates of disomy 21 in individual sperm samples from 0.00-0.44% [31-34, 36-38]. Interestingly, a correlation has been found between incidence of disomy 21 in spermatozoa and T21 in blood lymphocytes in both normal fertile controls and men suffering from subfertility [31, 33, 34]. Most often, however, only a single 21-specific probe has been used for the FISH analysis, diminishing the value of any results, due in particular to the risk of false positive signals [see *e.g. *12, 14].

It is also essential to note that there are a number of Case Reports in the literature, documenting paternal inheritance with either testicular T21 mosaicism identified *per se *or inferred from T21 mosaicism found in somatic tissues, most commonly blood lymphocytes. In addition, there are a number of reports demonstrating a raised incidence of disomy T21 sperm in fathers of T21 DS children in comparison to controls [Table 2 in 30]. These data indicate that a combination of germinal and somatic T21 mosaicism may be common among men in the general population.

### Somatic T21 Mosaicism

It is well known that children diagnosed as having DS by conventional cytogenetic analysis of a limited number of cells (usually 10-15 blood lymphocytes following *in vitro *culture) have a large number of clinical features, including a propensity for developing many different types of disease [review in 39-43]. To date a relatively high proportion of T21 Down syndrome fetuses are identified following chorionic villus sampling/amniocentesis, where again a small number of cells are analyzed. A recent large-scale study has highlighted that current screening procedures are incapable of differentiating between fetuses with an apparently normal karyotype in relation to those shown to be T21 mosaics by conventional cytogenetic analysis of amniotic fluid cells [44]. It is also well known that DS cases that have been suspected to be T21 mosaics based on subtle clinical features, and where therefore a larger number of cells have been analyzed, show a large variation in proportion of T21 cells [45-49].

The other side of this coin is the indication by a variety of studies that somatic T21 mosaicism in different tissues might not be uncommon in the general population, i.e. either in individuals with minimal DS features or indeed some subjects with no obvious clinical features of DS, including apparently normal fetuses, where termination has been performed for a non-medical/social reason [50, review in 14, 23, 51]. The respective phenotypes of subjects having this type of T21 mosaicism may reflect the percentage of T21 cells present in the different tissues [45].

One particularly interesting aspect of this situation concerns the potential effect as regards the etiology and pathogenesis of disease in the general population that occurs with an increased or decreased incidence among DS people. The outstanding question here is to what extent varying grades of T21 mosaicism in the relevant tissues might predispose or reduce the risk for people in the general population for these types of conditions. We here highlight this notion by reference specifically to childhood leukemias, solid cancers and Alzheimer’s Disease (AD).

It is essential to remember, however, that these examples might only constitute the tip of the iceberg. Thus, DS is also suggested to be a model for premature aging other than AD, and it may seem likely that T21 mosaicism is of importance for the etiology and pathogenesis of a range of common clinical conditions, such as immunodeficiency, infections, type 1 diabetes, hypothyroidism and asthma [see *e.g*. 23, 51-53]. Further large-scale investigations will be required to either substantiate or refute this thesis. Hopefully such studies, revealing factors shared in common between people diagnosed as having DS and those with the same condition in the general population will imply that new therapeutic strategies will be developed for the conditions in question.

#### T21 Mosaicism in Carrier Children may Increase their Risk of Developing Leukemia

Children diagnosed as having DS are particularly prone to develop two types of leukemia. Thus it is generally accepted that the incidence of Transient Acute Myeloid Leukemia (AML) is 350-500 times and that of Acute Lymphocytic Leukemia (ALL) 20 times more common in DS children than in children without any other overt phenotypic symptoms of DS [review in 54-60].

Much attention has during the last two decades been devoted to the role of an extra chromosome 21 in the development of childhood leukemias in DS in comparison to non-DS children [54-72]. In an initial study in 1990 Mitelman *et al. *recorded that T21 as an ‘acquired’ clonal chromosome change is common in hematological disorders and malignant lymphomas, but in most cases the extra chromosome 21 is present together with other numerical and/or structural changes. It was also concluded that the pattern of ‘acquired’ karyotypic changes is similar in patients with DS and in individuals with a normal constitutional karyotype [69]. Further studies have in the interim recovered both similarities and dissimilarities as regards details of the respective chromosomal aberrations [73].

Interest has recently focused on the molecular pathways in the multistep development of leukemias and it is generally accepted that ALL and possibly also AML originate during fetal hematopoiesis [74-83, review in 54]. To our knowledge there are to date no studies investigating the occurrence of T21 mosaicism in the relevant tissues in normal fetuses that could help to elucidate this notion. We suggest that it will now be essential to find out to what extent normal fetuses harbor T21 cells in the different tissues involved in fetal hematopoesis, i.e. the liver, thymus and spleen [review in 84], using a similar FISH approach as that described in *e.g*. Hultén *et al. *[24, 25, 30]. *A priori *it may seem likely that inter-individual tissue specificity in degree of T21 mosaicism underlies risk for children without any overt phenotypic features of T21 Down syndrome developing the different types of leukemia.

#### T21 Mosaicism may Protect Carriers Against Development of Solid Cancers

In sharp contrast to the situation as regards childhood leukemias, there are clear indications that people with DS have a substantially decreased risk of developing solid cancers, as recently highlighted by for example Sussan *et al. *[85]; Threadgill [86]; Yu *et al. *[87]; Baek *et al. *[88]; Gopalan *et al. *[89]; Patterson [43]; Tomlins *et al. *[90]; Fonatsch [54] and Ryeom *et al. *[91]. The exception to this general rule concerns childhood leukemias (discussed above) as well as a slightly increased rate of germ cell tumors in DS, especially testicular tumors. However, the increased risk of germ cell tumors might in fact be due to the high incidence of undescended testes in boys with DS [92, 93].

While a number of the epidemiological studies on DS and cancer come to slightly different conclusions regarding specific cancer types, the largest epidemiological study to date examined over 17,800 individuals with DS and found that mortality due to cancer (with the exception of leukemia and testicular cancer) is less than one-tenth of that expected in comparison to age-matched non-DS individuals [94]. Although there may be some bias in the interpretation of this and other studies, the protective anticancer effect of DS is significant. The lower incidence of nearly all cancers in individuals with DS implies that one or more of the trisomic genes on chromosome 21 exerts a broadly anti-neoplastic effect, presumably by modulating some common, fundamental aspect of tumor initiation and/or progression [91].

Recent work further suggests that the progression and expansion of tumors, not initiation, is the critical component of tumorigenesis that may be suppressed in DS [88]. Thus it is thought that expression of chromosome 21 genes beginning during embryogenesis in DS individuals allows the modest over-expression of these genes, which effectively prevent microscopic dormant tumors from undergoing an angiogenic switch [54]. Yet again, further work is required to find out to what extent T21 mosaicism in the respective tissues occurs during normal embryogenesis, and if so, how normal and T21 cells may interact. Hopefully further studies in this respect should allow deeper insight into the great *terra incognita *of cancer genetics [95] i.e. what constitutes tumor resistance, responsible for the protection of the majority of individuals against cancer development.

In addition, with the presence of at least four genes on chromosome 21 that function to negatively regulate angiogenesis by different mechanisms, it will also be of great interest to determine whether long-term, low-dose combination therapy with DSCR1, DYRK1A, endostatin and ADAMTS1 may offer broad cancer protection in all individuals and define a new modality of anti-angiogenic therapy [88, review in 54, 96].

#### T21 Mosaicism may Increase the Risk for Carriers to Develop Alzheimer’s Disease

One of the most characteristic clinical features of people diagnosed as having classical Down syndrome is symptoms of premature aging, including in particular the development of Alzheimer’s Disease (AD) at an early biological age [review in 97, 98].

Nearly two decades ago Huntington Potter suggested that chromosome segregation errors at cell divisions during embryonic development, leading to T21 mosaicism in different tissues, might underlie both disorders [99]. Much work has in the interim been devoted to the relation in origin between DS and AD, but the exact pathogenetic mechanisms are still not entirely clear [see *e.g*. 24, 25, 30, review in 100-105].

A number of authors have focused attention on T21 mosaicism in various tissues in non-DS patients suffering from AD as well as from age- and sex-matched controls in the general population. It is clear that there is an increased proportion of T21 cell nuclei in both blood lymphocytes and skin fibroblasts from non-DS patients diagnosed as having AD [106-110]. Also, women in some Alzheimer families in which the disease is inherited as an autosomal dominant mutation have given birth to a significantly higher than normal number of DS children [111-113]. It is of further interest that grandchildren of women with late onset Alzheimer’s Disease (LOAD) have been found to have an increased risk of developing the disease [114]. Most significant, however, is the observation of a substantially increased frequency (around 5- 10%) of T21 cell nuclei in relation to controls in brain tissue samples, ascertained following autopsy in AD patients [23, 98, 115].

Looking at the relationship from a DS perspective, it is of interest to note that there is as far as we are aware only one exception to the general rule that people with DS invariably develop AD prematurely. Remarkably, a 78 year old DS woman without any signs of AD was found to have an unusual chromosome set up, i.e. partial rather than regular T21 [116]. Furthermore, women without any overt clinical symptoms of DS, who have had a DS child already at a young age, develop AD at an earlier age than other women [29, 117, 118].

There are also a number of reports on people with none or minimal signs of DS, who have developed young-onset dementia of AD type, who have been found to have T21 mosaicism in peripheral blood samples. In the most recent Case Report [46] standard karyotype analysis from *in vitro *cultured blood lymphocytes revealed a level of 1/60 metaphase cells with T21, but a more extensive FISH analysis including interphase nuclei from uncultured blood cells identified a higher degree of T21 mosaicism, i.e. 20/200 (10%).

The outstanding question here is one of the ‘Hen and the egg’. In other words: Do AD patients have a *specific *predisposition for chromosome mal-segregation leading to somatic T21 mosaicism? Alternatively, might it be the other way round, where the question then is: Do AD patients, who do not show any clinically overt symptoms of DS have what we may call a ‘cryptic’ form of DS, characterized by T21 mosaicism, more or less specific to the brain?

## T21 MOSAICISM: HOW DOES IT COME ABOUT?

Many large-scale investigations (primarily family linkage analysis tracing DNA markers along the length of chromosome 21q between parents and DS children) have been devoted to the understanding of the origin of ‘complete’ T21 DS, concluding that the most important underlying factor is errors in maternal meiotic recombination of normal disomy 21 oocytes. This dogma has recently been challenged, proposing that parental T21 germinal mosaicism comprises an alternate mechanism that readily explains the situation including the well-known maternal age effect in DS [24, 25, 30].

By comparison much less attention has focused on the mechanisms underlying the origin of T21 mosaicism *per se*. In the early 1990s Antonarakis and co-workers concluded that the cause of T21 mosaicism, thought to affect around 2-4% of DS children, is embryonic mitotic chromosome mal-segregation, a process less affected by maternal age [119, 120]. Katz-Jaffe and co-workers have suggested that T21 mosaics diagnosed in early embryos (at the blastomere stage) would not be detected by standard analysis of amniocytes, and therefore all T21 fetuses diagnosed as such following amniocentesis would originate from T21 zygotes [121]. Assuming this diagnosis would include high-grade T21 mosaics, the vast majority might thus in fact originate by a so-called ‘meiotic’ error, this then followed by mitotic mal-segregation in the original T21 zygote [119-122]. Yet again, the situation as regards T21 mosaicism of intermediate degree (in the range of 5-95%) might be even more complex, as highlighted by Conlin *et al.* [122] using high resolution microarrays to identify the origin in patients with different types of chromosome aneuploidy mosaicism.

It is only recently that it has been discovered that low grade/cryptic T21 mosaicism is much more common than previously recognized, and the research in this area is still in its infancy. We have suggested that a more stringent control of mitotic segregation during early gonadal embryonic development may underlie the different degrees of T21 mosaicism in ovaries in relation to testicular samples [24, 30]. However, it is important to recognize that a combination of T21 germinal and somatic (so-called gonadal) mosaicism is not uncommon. Mal-segregation mechanisms involving both non-disjunction and anaphase lag are likely to occur during the different stages of development in different tissues from the embryonic and fetal stages into adulthood, and more research in this area is required to come to grips with the relative influence of either of these mechanisms in the generation of the different types of tissue-specific T21 mosaicism [1, 23, 123, 124]. One way to further our understanding in this respect would be investigations recording the phenomenon of uniparental disomy (UPD). Thus, in the absence of somatic recombination, cases of T21 mosaicism originating from post-zygotic mal-segregation in an original normal disomy 21 zygote would be expected to be isodisomic for two of the three chromosomes 21, making up this somatic acquired aneuploidy [reviews in 122,^ 124^-^127^].

Finally, it will be of added interest to find out more about the relation between the different types of conditions that may be associated with T21 mosaicism, asking questions such as (1) whether or not there is any indication of a reduced risk of solid cancers in people suffering from AD [128], and (2) whether or not T21 mosaic women ascertained because of early onset AD [45] have an increased risk of T21 DS conceptions. Further work is also required to find out more about the potential role of environmental factors in this regard [27, 129-133].

## SUMMARY AND CONCLUSIONS

It is now nearly 50 years since the first cases of T21 mosaicism was recorded [134-139]. In the interim it has become well established that a number of people diagnosed as having the typical DS phenotype are T21 mosaics with a small proportion of cells in lymphocyte cultures having the normal chromosome constitution. It has also become increasingly clear that some people with minimal signs of DS and indeed some without any obvious such DS signs are low grade/cryptic T21 mosaics with respect to various other tissue samples [50, review in 14, 23, 51]. The identification of low grade/cryptic T21 mosaicism is, however, hampered by technological problems. Thus, to date it is only the application of FISH technology with a number of chromosome-specific probes on large cell populations from the respective tissue samples that allows identification of this type of subtle chromosome abnormality (Figs. **2, 3**) [review in 9-19, 22, 23].

Considering the labor intensity and costs together with the problems as regards access to the relevant tissue samples, it is perhaps not surprising that our knowledge in this field is still quite limited. This concerns in particular germinal mosaicism, where it is only recently that it has become clear that most if not all female fetuses may in fact be germinal T21 mosaics. On the other hand, a very much lower incidence of germinal T21 mosaicism has been found in male fetuses. On the basis of these observations we suggest that the chance of a T21 conception may be largely related to incidence of fetal germinal mosaicism in individual males and females, and the DS maternal age effect may be due to accumulation of T21 oocytes from fetal life until ovulation [24, 25, 30 and Hultén *et al. *unpublished observations].

The situation as regards somatic T21 mosaicism is even more complex, and to date it is only a restricted number of somatic tissues in a limited number of subjects that have been investigated regarding this character [50, review in 14, 23, 51]. Most studies have been initiated with a view to test the hypothesis that T21 mosaicism might underlie a specific clinical condition. The main conclusion from these studies is that T21 mosaicism in the various somatic tissues is much more common than has been previously recognized, and further studies are required to get to grips with its origin.

A number of studies have shown that there is an association in this respect between conditions in DS and the variants of the same condition in the non-DS general population. We have here highlighted this notion as regards three specific clinical conditions, childhood leukemias, solid cancers and Alzheimer´s Disease. It seems likely that T21 mosaicism in the respective tissues plays a role in the pathogenesis in a range of other conditions that are more common among DS people than non-DS. Further work in this area is likely to allow development of not only more efficient therapy *per se *but hopefully also the introduction of broad protection to all relevant individuals. It will in this regard be of particular interest to identify suitable biomarkers to allow differentiation between those people that are at high risk in relation to those at low risk for the different conditions in question. Perhaps we should to this effect look again at the potential utility of such soft signs as the typical dermatoglyphics, as first so elegantly documented by Penrose more than half a century ago (Fig. **1**) [4, 5, 7, 8, 140, 141].
